# Why and How European Farmers Are Dedicated to Breeding the Dwarf Dahomey Cattle

**DOI:** 10.3390/ani12030377

**Published:** 2022-02-04

**Authors:** Sèyi Fridaïus Ulrich Vanvanhossou, Sandrine Odounyèmi Houessou, Kathrin Halli, Isabella Jasmin Giambra, Kerstin Brügemann, Luc Hippolyte Dossa, Sven König

**Affiliations:** 1 Institute of Animal Breeding and Genetics, Justus-Liebig-University Gießen, 35390 Gießen, Germany; kathrin.halli@agrar.uni-giessen.de (K.H.); isabella.j.giambra@agrar.uni-giessen.de (I.J.G.); kerstin.bruegemann@agrar.uni-giessen.de (K.B.); sven.koenig@agrar.uni-giessen.de (S.K.); 2 School of Science and Technics of Animal Production, Faculty of Agricultural Sciences, University of Abomey-Calavi, Abomey-Calavi 03 BP 2819, Benin; houessouo.sandrine@yahoo.fr (S.O.H.); hippolyte.dossa@fsa.uac.bj (L.H.D.)

**Keywords:** African shorthorn taurine, small-sized cattle, grassland values, breeding systems, smallholder, resistance to diseases, Benin

## Abstract

**Simple Summary:**

The introduction of high productive cattle breeds in Africa is well known, but the contribution of African breeds to livestock biodiversity in Europe is generally overlooked. This study reports, for the first time, European farmers’ interests in keeping the Dahomey cattle, and characterizes their management practices. The Dahomey cattle from Benin (West Africa) are the smallest cattle in the world, and they were introduced to Europe in the early 1900s. The findings revealed that European farmers are increasingly interested in keeping Dahomey cattle, because of their suitability for grassland maintenance and meat production, as well as their low management requirements (with regard to feeding, preventive and curative health care and reproduction management). Overall, the study displays the agricultural importance and ecological utilization of Dahomey cattle in European countries. It shows how small-sized cattle can support the promotion of sustainable livestock production and the management of ecosystems, including faunistic and floristic diversity.

**Abstract:**

This study investigates the motivations and breeding practices of farmers keeping Dahomey cattle in European countries. Data were collected using a web-based open-closed questionnaire survey targeting 55 farmers from Germany, Switzerland and Austria. Descriptive analyses revealed that the earliest European Dahomey herds were established in 2005. Moreover, interest in the breed recently increased as 63.7% of the investigated farmers established their herds between 2016 and 2020. The average herd size comprises seven Dahomey cattle, kept for managing grassland (59.3%), for production of meat or as breeding stock (32.1%) and for a hobby (8.6%). The animals are mostly kept in grazing systems throughout the year, partly fattened with supplement feeds. The low disease incidence and no need for extra health care in the herds indicate the robustness of the breed. Furthermore, meat quality, calving ease, small size, calm character and low feed requirements of Dahomey cattle were valued by the farmers. For the preservation of these features, farmers confirmed their enthusiasm to support any breeding and conservation program of this smallholder breed in Europe and Benin. This study highlights the importance of small-sized cattle for sustainable breeding systems and with regard to ecosystem management practices.

## 1. Introduction

**Figure 1:**
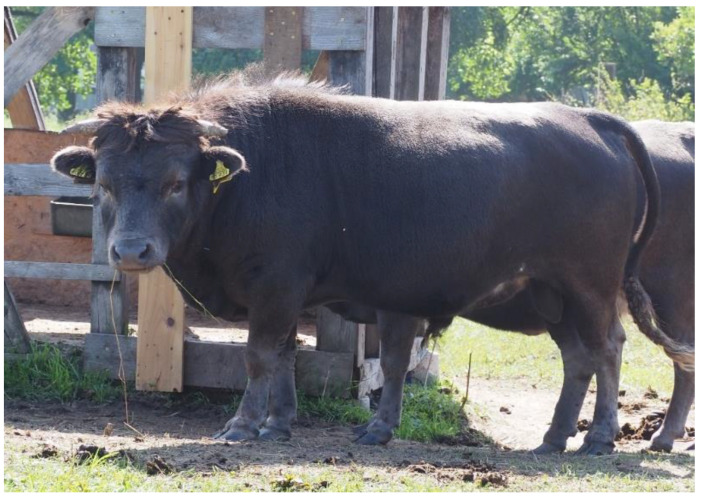
A three years old Dahomey bull kept in Germany.

Dahomey is the former name of the Republic of Benin in West Africa. This name is also attributed to the smallest cattle worldwide, originating from Benin [1]. The Dwarf Dahomey cattle are characterized by an average wither height of 90 cm with an average adult body weight of 180 ± 30 kg for cows and 260 ± 40 kg for bulls (Figure 1) [1,2]. The small size of these shorthorn taurine (*Bos taurus*) animals is associated with easy calving, and with several favorable features including adaptation to marginal areas and resistance to diseases [1,3]. Dahomey cattle, known as Lagune cattle in Benin, are adapted to tropical environments through their original distribution to the rainforest and coastal regions of western and central Africa [4]. They are kept in Benin in challenging production systems for multiple purposes including socio-cultural functions (social status, sacrifices, bride wealth), draught (integration of livestock and crop production), means of saving or insurance and meat production, but not for milk production [4,5]. Unfortunately, the Lagune breed is increasingly threatened in Benin by indiscriminate crossbreeding with large-sized animals, and the lack of appropriate strategies or policies to promote and ensure its sustainable use [6]. In addition, the breed is poorly investigated and no information exists with regard to their population size in other African countries or in Europe [7]. Recent genomic characterization using medium-density SNP chips confirmed the Beninese indigenous Lagune breed as the closest ancestor of Dahomey cattle kept in Europe [8]. Similarly, applying phylogenetic analysis of the mitochondrial D-loop DNA sequence, Pfeiffer et al. [9] observed a large genetic distance between the Dahomey cattle and European taurine breeds. 

Dahomey cattle were introduced in diverse African (e.g., Gabon, Democratic Republic of Congo, Zambia) and European countries in the early twentieth century [4,9]. According to existing reports, the Antwerp zoo in Belgium was the first destination of Dahomey cattle in Europe [2]. To date, the breed is kept in several zoos and private herds in European countries [9]. The *Zootierliste* website [10] indicated 39 zoos in Europe keeping Dahomey cattle, including 23 zoos in Germany, five zoos in the Czech Republic and three zoos in Switzerland, two in The Netherlands, two in Austria, two in France, one in Belgium and one in Hungary. European farmers are organized in diverse breeding associations, including the *Verband Europäisches Dahomey-Zwergrind* (VEDZ; European Association for Dahomey Dwarf Cattle) [2]. This association, founded in 2001, currently includes 77 breeders from Germany, Austria, Switzerland and the Czech Republic [2]. In addition, further national associations of Dahomey cattle breeders include the *Verein Dahomey Schweiz* in Switzerland and the *Dahomey Zuchtverband Deutschland e.V.* in Germany [11]. The later association, the most recent to our knowledge, was founded in July 2020 [11].

The diversity of Dahomey cattle herds and breeding associations in Europe reflect an increasing interest in the breed. The VEDZ reports an ongoing application procedure for the registration of the breed in the German catalogue for cattle breeds. Young animals or reproducers of Dahomey cattle are regularly marketed, also on online platforms. Yet, the breed is barely addressed in scientific studies. Golze [12] recently compared meat quality traits of 11 cattle breeds in Germany and reported higher organoleptic characteristics (including tenderness and special flavor) for Dahomey cattle. In addition, the meat of Dahomey has valuable nutritional content, because of the high proportion of absorbable iron and the low cholesterol content. Similar valuations for Dahomey cattle meat are reported on the website of several breeders and breeding associations [11,13,14,15]. Golze [12] related the appreciable nutritional characteristic of Dahomey cattle meat with the low growth rate of the animals and the feeding system characteristics. Moreover, the adaptation of small-sized cattle to marginal or harsh production systems is well acknowledged [1]. In the ongoing global context of climate change, the Dahomey cattle may represent a good alternative for sustainable cattle farming under limited resources [1]. Conventional livestock production systems are increasingly reported worldwide for their negative impacts on the environment, including the degradation of grasslands (i.e., vegetation communities dominated by herbaceous) [16]. In Europe, grasslands are largely reliant on regular removal of aboveground biomass by agricultural land use, including grazing and/or mowing [17]. Grassland maintenance practices aiming at conserving floristic biodiversity and enhancing the sustainability of land ecosystems are therefore required. European agricultural policy and researchers recommend low pressure on grasslands with seasonal pastures, and the use of specific animal species or breeds presenting specific grazing behavior or low feeding requirements [16,18,19]. Hence, the Dahomey breed should be promoted, stimulating scientific investigations and political support in this regard. The current study characterizes for the first time the breeding systems of the Dwarf Dahomey cattle in Europe. Farmer interests, their satisfactions and suggestions for the promotion of this breed are subsequently addressed.

## 2. Materials and Methods

### 2.1. Data Collection

An open-closed questionnaire focused on socio-economic characteristics, the interest of farmers in keeping Dahomey cattle, the establishment of Dahomey herds and the membership in breeding associations, acquisition of initial herds (origin, price of the animals), initial and current herd sizes and herd composition, management system characteristics and management constraints, animal exploitation and performances, as well as farmer suggestions for the promotion of Dahomey cattle. Socio-economic characteristics included the farm location, age of farmers and their professional activity and experience in cattle breeding. Herd composition comprised the respective number of calves, bulls and cows, and the presence of other breeds in the herd. The herd management system was surveyed with regard to housing, feeding, reproduction and health management practices. The performance evaluation of animals included body weight and the female fertility traits age at first calving and calving interval. As Dahomey cattle are not used for milk production, no records were available for milk or protein yield. However, farmers were asked to rate milk production, animal growth, meat quality and fertility of Dahomey cattle using the grades “high”, “reasonable”, “low”, “very low”. In addition, farmers categorized their appreciation (i.e., “like”, “do not like” or “no appreciation”) for specific characteristics of Dahomey cattle including the calm character, calving ease, feeding requirements, disease resistance and the small body size. Finally, the farmers indicated their overall satisfaction with the Dahomey breed with a grade between 5 and 1 (5 = highly satisfied, 1 = not satisfied).

The questionnaire (see data availability section) was prepared on the “Google Form” online platform and submitted to the members of the VEDZ (77 members in total) [2] and the “*Verein Dahomey Schweiz*” (50 members in total) between Mai and October 2021. Further known Dahomey breeders without a breeding organization membership, were invited. A total of 55 farmers voluntarily participated in the survey. They represent 43% of the members of the surveyed breeding associations.

### 2.2. Data Analysis 

The data were analyzed with basic R packages including *dplyr* and *tidyr* [20,21], and using elementary descriptive statistics, i.e., frequency distributions for qualitative variables and means for quantitative variables. All the graphs were prepared with the *ggplot2*, *viridis*, and *viridisLite* packages [22,23,24].

## 3. Results

### 3.1. Socio-Economic Characteristics and Motivations of the Farmers

**Figure 2:**
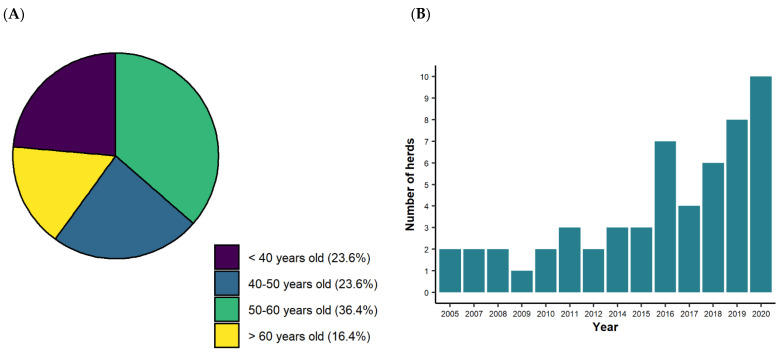
(**A**) Distribution of the investigated European Dahomey farmers according to age groups; (**B**) Number of established Dahomey cattle herds per year between 2005 to 2020 in Germany, Austria and Switzerland, indicating the development of European farmers’ interest in the Dahomey cattle breed.

The majority of the respondents resided in Germany (60.0%), followed by Switzerland (36.4%) and Austria (3.6%). They were mainly men (72.7%) and were in majority between 40 and 60 years old (Figure 2A). Most of Dahomey holders (64.2%) were not professional farmers, and a fraction of 94.3% was professionally active in an off-farm occupation. Farmers’ experience in cattle breeding varied between one and 16 years. Two farmers breed Dahomey cattle since 2005. A total of 63.6% of the respondents adopted the breed between 2016 and 2020 (Figure 2B). In addition, 85.5% of the respondents are members of a breeding association, and 69.6% of them received their membership during the past five years. Motivations for holding Dahomey cattle included grassland maintenance (59.3%), reproduction and meat production (32.1%) and hobby farming (8.6%).

### 3.2. The Dahomey Cattle Herds

#### 3.2.1. Acquisition of Initial Stocks: Origins and Prices 

**Figure 3:**
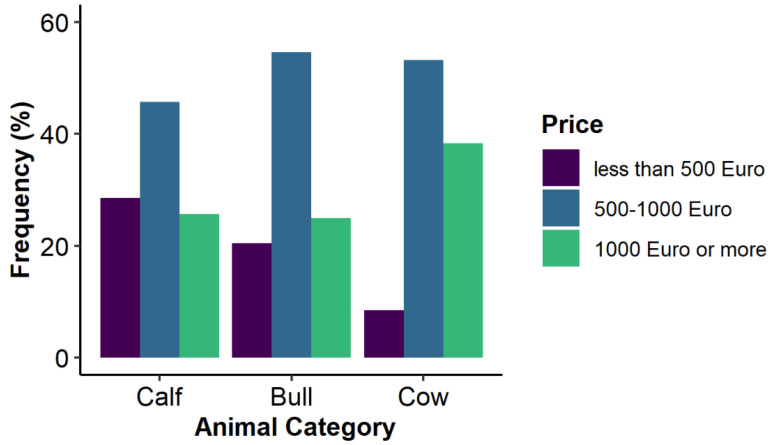
Acquisition price of Dahomey cattle for calves, bulls and cows in Germany, Austria and Switzerland. Calf = cattle less than one year old; bull and cow = reproductive male and female cattle, respectively.

Dahomey farmers established their initial breeding stock with one to 10 animals (three animals on average). The animals were mainly acquired from fellow farmers (69.1%), through the VEDZ (21.8%) or on online platforms (5.5%). Only two farmers (3.6% of the respondents) acquired their initial Dahomey cattle from zoos, namely the *Tierpark Brüggen* and *Zoo der Minis Aue e.V.* Figure 3 displays the distribution of the acquisition price. The majority of the animals were purchased for 500 to 1000 Euros. Cows represented the most expensive category, costing more than 1000 Euros.

#### 3.2.2. Herd Sizes and Composition

**Figure 4:**
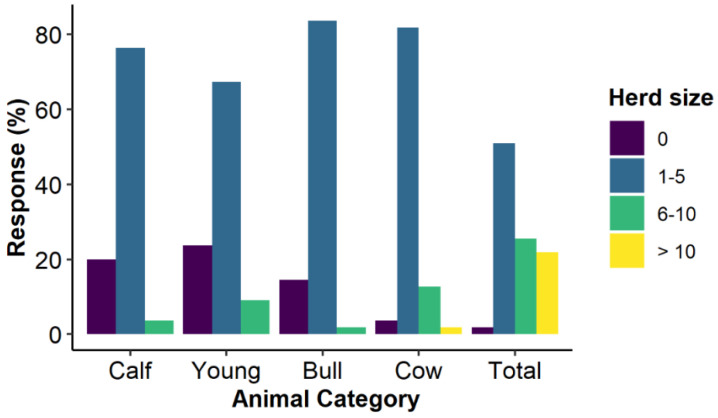
Herd size and structure of the investigated Dahomey cattle in Germany, Austria and Switzerland. Calf = cattle less than one year old, young = cattle between one and three years old, bull and cow = reproductive male and female cattle, respectively.

Dahomey cattle herds consisted of two to 23 Dahomey animals, with an average of seven animals per herd. Cows and bulls were the predominant categories. Calves and young cattle were absent in more than 20.0% of the herds (Figure 4). A total of 83.6% of the investigated herds only consisted of Dahomey cattle. The remaining herds (16.4%) included additional breeds such as Angus, Belgian Blue, Limousin and crossbreeds with Dahomey.

### 3.3. Herds Management

#### 3.3.1. Housing and Feeding 

The housing and feeding systems of Dahomey cattle varied according to seasons (Table 1). Animals were mainly kept on pasture or in open barns. The open barn system with protections against cold floor, snow and rain using windbreak were the predominant housing mode in winter. In 58.2% of the investigated herds, access to pasture was combined with open or closed barns during all seasons. A fraction of 34.6% of the respondents kept Dahomey cattle on pasture during the whole year, apart from the winter months. 

In addition to the utilization of natural grazing systems, animal feeding in Dahomey herds included own produced or purchased feed and supplements (Table 1). Own produced feed was the most prevalent feeding component in winter. Feeding strategies in Dahomey cattle focused on grassland maintenance through free and rotational grazing (74.6%), but also to improve meat production (25.5%). 

#### 3.3.2. Reproduction and Health Management

Natural mating was very common in Dahomey cattle herds. A small fraction of 5.5% of farmers used artificial insemination accomplished by veterinarians or technicians. Nevertheless, three-fourths of the respondents selected herdbook bulls (76.4%) and registered their animals in herdbooks (74.6%). Castration of bulls was applied in 45.5% of the herds during the first or second age year.

**Figure 5:**
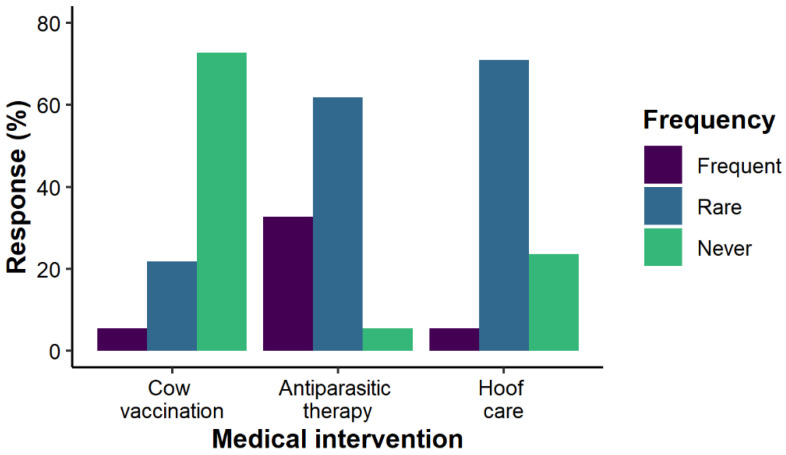
Frequency of medical interventions in Dahomey cattle herds from Germany, Austria and Switzerland.

A quite large fraction of 32.7% of the respondents indicated the absence of frequent animal diseases. Nevertheless, diarrhea was the most reported disease symptom (in 40.0% of the investigated herds). Further rare diseases included pneumonia, coccidiosis and parasitic infestations. Almost all Dahomey farmers (92.7%) controlled the health status of their animals once per day. Curative treatments were generally rare in Dahomey cattle (Figure 5). Therapies against parasitic infestations were the most frequent in the herds (32.7% of the respondents), while hoof care was very rare or has never been applied in 94.6% of the investigated herds. Similarly, 72.7% of the Dahomey farmers have never vaccinated their animals. Moreover, a particular or sudden case of animal death has never been experienced in 92.7% of the investigated herds. 

#### 3.3.3. Animal Exploitation

Dahomey cattle were slaughtered frequently and occasionally in 7.3% and 45.5% of the surveyed herds, respectively. Forty percent of the surveyed farmers reported an average of one to four animals slaughtered per year. Meat production (5.5% of the respondents) and herd management factors (40.0% of the respondents) were the main reasons for animal slaughter. The later factors included reduction of the herd size, impossibility to sell the animal, infertility or availability of another bull, animal character (e.g., aggressive), age and health status. Dahomey animals were generally slaughtered between their second and third year of life (29.1% of the respondents). 

The majority of investigated farmers reported that they sold Dahomey reproducers frequently (30.9%) or occasionally (41.8%). Animal prices varied according to sex, and Dahomey cows generally valued more than 1000 Euros (51.2% of the respondents). In contrast, only 30.2% of the respondents described such a price range for bulls. According to 51.2% of the farmers, the price for Dahomey bulls ranged between 500 and 1000 Euro.

#### 3.3.4. Animal Performances and Farmers’ Valuations

**Figure 6:**
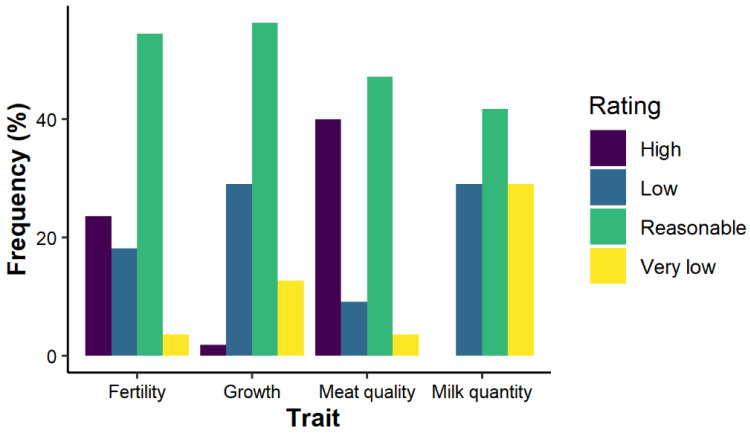
The ratings of European farmers for the Dahomey cattle performances.

Meat quality and cow fertility in Dahomey cattle were highly rated by 40.0% and 23.6% of the Dahomey farmers, respectively (see Figure 6). In contrast, 58.2% of the surveyed farmers reported low or very low milk performance in the breed. In contrast, 56.4% of the respondents rated Dahomey cattle growth (body weight gain) as reasonable. The average adult weight of Dahomey cows (> two years old) ranged between 200 and 300 kg (65.5% of the respondents), while adult bulls weighed 300 kg on average (according to 36.4% of the farmers). 

In 80.0% of the investigated herds, the age at first calving of cows was between 24 to 36 months. Nevertheless, some farmers (18.2%) reported an age at first calving below 24 months. In most cases (70.9% of the respondents), the average calving interval was about one year. Calving was generally year-round in the majority of the herds (52.7%), but was more frequent in spring and autumn (36.4%) or summer (9.1%) in some herds. 

Overall, 60.0% and 32.7% of the respondents rated the performance of Dahomey cattle with the satisfaction grade of 5 and 4, respectively. Traits mainly appreciated in the Dahomey cattle included the calving ease (100% of the respondents), resistance to diseases (98.2% of the respondents), small size (94.6% of the respondents), calm character (83.6% of the respondents) and low feed requirements (70.9% of the respondents). 

### 3.4. Promotion of the Dahomey Cattle and Breeding Constraints 

Farmers expressed their willingness to support eventual breeding programs in Dahomey cattle in Europe (85.5%) and in Benin (72.7%). They recommended selection strategies in Dahomey cattle focusing on meat quality and quantity (81.8% and 49.1% of the respondents, respectively). However, 29.1% of the farmers emphasized the necessity to preserve the originality of the breeds including the small body size, female fertility, robustness, resistance to diseases and coat color. Moreover, the farmers suggested the promotion of the Dahomey cattle mainly through advertisement (52.7% of the respondents) and the reinforcement of breeding associations and partnerships (7.3% of the respondents).

Most of the surveyed farmers (61.8%) reported no constraints with regard to breeding aspects of Dahomey cattle. The few constraints identified by some farmers included the shortage of grazing land, the high slaughter costs, the aggressiveness of some bulls, the lack of insemination facilities, and the limited support from agricultural authorities. In addition, some farmers complained about the consideration of Dahomey cattle as one livestock unit (LU) like larger cattle breeds.

## 4. Discussion

### 4.1. The Interests of European Cattle Breeders in the Dahomey cattle 

The current study identified an appreciable number of farmers interested in keeping Dahomey cattle, although they represent a minor fraction of the overall beef farmers in Europe (e.g., 49,675 beef farmers in 2021 in Germany) [25]. A large number of Dahomey farmers from Germany may be related to the creation of the breeding association (VEDZ) 20 years ago in the country [2]. Moreover, the presence of Dahomey cattle in 23 zoos in Germany enhanced the breed attractiveness [10]. The oldest Dahomey cattle herd reported in this study was established in 2005. However, due to the establishment of the VEDZ in 2001 [2], earlier Dahomey cattle herds may exist. Additionally, Dahomey breeders from Austria and Switzerland are reported in this study. The young breeding association (*Verein Dahomey Schweiz*), established in Switzerland two years ago, reflects the numerous farmers investigated in this country. Further Dahomey cattle farmers are located in other European countries including the Czech Republic [2], but they did not participate in this survey. The increasing number of farmers adopting Dahomey cattle, as observed in the past five years, indicates a further expansion of the Dahomey cattle population in the next years. 

The findings revealed that Dahomey cattle are mainly kept for grassland maintenance. The suitability of the breed for grassland maintenance is due to the small body size and low feed requirement [1]. The Glanrind, Rotes Höhenvieh and Limpurger breeds are similarly reported for grassland maintenance in Germany [26,27]. However, in contrast to Dahomey, these breeds are characterized by decreasing population size in Germany [26,27]. Cattle husbandry in extensive grazing systems is described as a sustainable nutritional strategy to reduce the negative impacts of livestock production on the environment [28]. Researchers described an increasing enthusiasm for extensive cattle grazing systems in European countries [29,30]. For instance, Bunzel-Drüke [31] reported an “all-year grazing project” with Heck cattle for the sustainable management and the conservation of the natural grassland in Central Europe. According to Schley and Leytem [29], cattle breeding for grassland maintenance offers outstanding convenience for the sustainable management of ecological niches and the promotion of faunistic and floristic diversity. Likewise, Tóth et al. [30] observed that cattle grazing induces a more species-rich and trait-rich vegetation with the higher cover of forbs, in comparison to sheep grazing. Sheep have a highly selective grazing behavior, and they are more sensitive to environmental conditions affecting pasture quality and grazing time than cattle [32]. The availability of small-sized Dahomey cattle that are suitable for grassland maintenance is therefore a strong advantage in this regard. In addition, the Dahomey cattle breed fulfills further breeding objectives including meat production. Cattle grazing systems will be promoted in European countries such as Germany, because of the higher consumption of cattle meat compared to sheep meat [33]. One-third of the investigated farmers confirmed that they use Dahomey cattle for meat production. Similarly, multi-purpose use is known for the Rotes Höhenvieh cattle breed [27]. Rotes Höhenvieh cattle are of medium size and able to produce under harsh environments, similarly to Dahomey cattle [34]. 

The interest in breeding medium or small sized cattle is not uncommon. Several small sized cattle breeds attracted farmers and scientists worldwide [35]. For instance, the Dexter, small cattle from Ireland, are kept and preserved as purebred. Other cattle breeds including the miniature Hereford, Lowlines (small Angus cattle), Jersey or Zebus are exclusively selected for their small size [35]. According to Boden [35], reasons to keep small sized cattle include their easier management requirements, i.e., regarding feeding (pasture), housing, labor time, etc. [35]. This observation is in line with the dominance of non-professional farmers (with off-farm occupation) in the sampling design from the present study. In addition, more than 50.0% of the surveyed farmers were more than 50 years old, which concurred with the appropriateness of small sized cattle for elderly people seeking to invest in cattle [35]. Because the Dahomey cattle require low management resources, they represent a convenient option for future cattle farming systems [1]. Indeed, the increasing scarcity of agricultural resources (e.g., land) in several areas is well acknowledged [35]. Accordingly, small sized cattle are promoted for local urban farming or challenging environments (e.g., extreme temperatures) [35]. These observations may stimulate researchers and policy makers, especially in Benin, to preserve and promote indigenous cattle breeds despite their small body size.

### 4.2. Breeding Systems for Dahomey Cattle in European Herds

The management systems of Dahomey cattle in the investigated herds are in accordance with their main production objectives (i.e., grassland maintenance, meat production), and their robustness. Dahomey cattle are mainly kept in small herds (seven animals on average) under extensive grazing systems all year round, and with almost no health preventions and treatments. In addition, reproduction management mainly involves natural mating with less intervention from veterinarians. The management system of Dahomey cattle is comparable with the one of the Lagune (the ancestor of the Dahomey cattle) in Benin. Similar management systems were observed in European cattle herds targeting beef production, grassland maintenance, hobby farming or organic cattle breeding [29]. Accordingly, a surveyed farmer from Germany favored Dahomey cattle breeding for organic production systems. Consequently, the Dahomey breed is a valuable alternative for organic farming, which is continuously increasing in Europe. 

Animal slaughtering and sale of breeding stock indicate the economic importance when keeping Dahomey cattle. The associated costs are quite small, due to the low management requirements. The rarity of diseases as well as the lack of proper preventive and curative health care corroborate the disease resistance of the breed [3]. The exchange of Dahomey cattle, mainly among fellow farmers, and the small population size of the European Dahomey cattle, are reasons for the high genomic inbreeding [8]. The inclusion of reproducers from zoos may reduce inbreeding in the population and increase the effective population size. Preliminary evaluation of the Dahomey cattle population kept in zoos is imperative in this regard. Moreover, the willingness of several farmers to register their animals in herdbooks is a relevant opportunity to manage genetic diversity in the Dahomey population [8]. Pedigree information from herdbooks is also beneficial for establishing breeding strategies and preserving the breed.

### 4.3. Promotion of Dahomey and Lagune Cattle Breeds 

The reported performances of the Dahomey cattle are in line with the breed valuations by the farmers. The age at first calving between two and three years, as well as the calving interval of one year, illustrate the high fertility status of the Dahomey cows. Similar reproductive performances are reported for Lagune cattle in Benin [5]. Likewise, for several cattle breeds kept in Germany [36,37], the age at first calving and calving interval varied between 24 to 36 months, and 361 to 380 days, respectively. Moreover, the average adult weight of about 300 kg concurs with the reasonable growth and low feed requirement of this breed, as known for Lagune cattle [38]. Despite their low average body weight, farmers reported that Dahomey cattle are categorized using the standard LU system (like large cattle breeds). Indeed, German agricultural policies attribute LU coefficients to animals based on their species, age and sex, whereby an adult cow and bull (over two years old) is equivalent to 1.2 LU, a heifer (female young cattle between one and two years old) represents 0.6 LU, a bull-calf (between one and two years old) represents 0.7 LU and a calf (female or male up to one year) represents 0.3 LU [39]. Yet, an adult Dahomey cattle (with an average body weight of 300 kg), would correspond to 0.6 LU following the scientific definition of one LU, which is equivalent to 500 kg body weight [40,41]. Against this background, the revision of classification systems for LU will support the promotion of small size cattle breeds like Dahomey.

In addition to the easy feeding management of Dahomey cattle, most of the surveyed farmers valued the meat quality. Comparable valuation for meat quality is reported for Beninese local breeds and the Rotes Höhenvieh, which are kept in similar grazing systems in Germany [42,43]. The phenotypic performances in a favorable range of Dahomey cattle justify the overall satisfaction of the farmers and their enthusiasm to promote this breed. In this regard, the current organization of the farmers in breeding associations definitely will facilitate breeding program improvements. Lagune breeders in Benin may benefit from the rich experience of the Dahomey breeding associations to Lagune cattle. For instance, the creation of collaborations between the VEDZ and Beninese farmers will contribute to knowledge exchange. Such programs should be initiated because the majority of the investigated Dahomey farmers confirm their enthusiasm with regard to the promotion of the Dahomey breeds in Benin and Europe. The current study constitutes a good starting point for such purpose, as it increases the visibility of the breed as suggested by the farmers.

## 5. Conclusions

Dahomey cattle have a great potential to be promoted in European countries because of their suitability for grassland maintenance. The association of cattle breeding with ecosystem management is of increasing importance, especially due to the ongoing global environmental challenges. Moreover, the easy management of the Dahomey cattle, associated with their disease resistances, their fertility and their meat quality are very valuable for cattle farming in small-sized herds, including meat production. Despite their importation to Europe, the breeding systems and performances of Dahomey cattle are similar to those of their ancestors, i.e., the Lagune, in Benin.

## Figures and Tables

**Table 1:** Housing and feeding management systems in different seasons in the Dahomey cattle herds from Germany, Austria and Switzerland.

	Season			
	Winter	Spring	Summer	Autumn
**Type of housing (%)**				
Open barn	53.6	36.0	31.1	34.7
Only on pasture	26.1	54.7	62.2	58.3
Closed barn	20.3	9.3	6.8	6.9
**Type of feeding (%)**				
Pasture	18.7	46.0	58.4	50.5
Own produced feed	52.8	36.0	25.8	31.6
Purchased feed	14.3	9.0	5.6	8.4
Supplement	14.3	9.0	10.1	9.5

## Data Availability

The raw data supporting the results of this article are stored at the server of the University of Giessen and will be made available upon reasonable request in accordance with data protection regulations.
